# Coffee Consumption and Risk of Fracture in the Cohort of Swedish Men (COSM)

**DOI:** 10.1371/journal.pone.0097770

**Published:** 2014-05-15

**Authors:** Helena Hallström, Alicja Wolk, Anders Glynn, Karl Michaëlsson, Liisa Byberg

**Affiliations:** 1 Department of Surgical Sciences, Section of Orthopaedics, Uppsala University, Uppsala, Sweden; 2 Institute of Environmental Medicine, Division of Nutritional Epidemiology, Karolinska Institutet, Stockholm, Sweden; 3 Risk and Benefit Assessment Department, National Food Agency, Uppsala, Sweden; Loyola University Medical Center, United States of America

## Abstract

**Background:**

Recent research in a large cohort of women showed that coffee consumption is not associated with increased risk of fracture. Whether this is the case also among men is less clear.

**Methods:**

In the Cohort of Swedish Men (COSM) study, 42,978 men aged 45–79 years old at baseline in 1997 answered a self-administered food frequency questionnaire covering coffee consumption and a medical and lifestyle questionnaire covering potential confounders. Our main outcomes first fracture at any site and first hip fracture were collected from the National Patient Registry in Sweden. The association between coffee consumption and fracture risk was investigated using Cox’s proportional hazards regression.

**Results:**

During a mean follow-up of 11.2 years, 5,066 men had a first fracture at any site and of these, 1,186 (23%) were hip fractures. There was no association between increasing coffee consumption (per 200 ml) and rate of any fracture (hazard ratio [HR] 1.00; 95% confidence interval [CI] 0.99–1.02) or hip fracture (HR 1.02; 95% CI 0.99–1.06) after adjustment for potential confounders. For men consuming ≥4 cups of coffee/day compared to those consuming <1 cup of coffee/day, HR for any type of fracture was 0.91 (95% CI 0.80–1.02) and for hip fracture: 0.89 (95% CI 0.70–1.14).

**Conclusions:**

High coffee consumption was not associated with an increased risk of fractures in this large cohort of Swedish men.

## Introduction

Osteoporotic fractures - the ultimate manifestation of osteoporosis - are affecting a growing number of elderly individuals globally [1]. Both men and women are affected by osteoporosis, but despite a lower risk of osteoporotic fractures in men, the morbidity and mortality seem to be greater in men having experienced such fractures [2].

A number of dietary factors have been discussed in the aetiology of osteoporosis, including consumption of caffeine-containing beverages [3]–[6], especially coffee, which has a relatively high concentration of caffeine [7]. Some studies have demonstrated an association between caffeine intake and calcium homeostasis in humans [8], [9] and negative effects on osteoblast function in vitro [10]–[12]. Epidemiological studies investigating the relation between coffee, tea consumption and caffeine intake and the risk of fractures are fairly abundant in women but scarce in men. Results from the three previous cohort studies in men have shown no association [13], [14], and a decreased risk of fracture [15], also summarized in a recent meta-analysis [16].

The incidence of fractures is high in Sweden, also among men [17]. In an international comparison intake of coffee (and thus intake of caffeine) is similarly high in Sweden [18]. Thus, studying the relation between coffee consumption and the risk of fractures in Sweden may be optimal [19]. We recently published results from the so far largest epidemiological study concerning coffee consumption and fracture risk in women. We found that whereas a high coffee consumption is associated with slightly lower bone mineral density (BMD), this is not manifested in an increased risk of fracture [20]. We have also previously demonstrated an association between high coffee consumption and a decrease in bone mineral density (BMD) in older men [21]. Importantly, however, fractures in elderly are not only the consequence of osteoporosis but factors related to the risk of falling are also of importance [22], [23].

The primary aim of this investigation was to study the association between coffee intake and the risk of incident fractures in a large prospective population-based cohort of Swedish men 45–79 years old at the beginning of the study. A secondary aim was to evaluate whether risk of fracture in relation to coffee consumption was affected by calcium intake.

## Methods

### Study Population

The Cohort of Swedish Men (COSM) was created in the autumn of 1997 [24]. All male residents (n = 100,303, aged 45–79 years) of Örebro and Västmanland Counties in central Sweden were invited to participate in the study. Along with the invitation, they received written information about the study and a self-administered questionnaire that included almost 350 items on diet and other lifestyle factors (e.g., socio-demographic data, waist and hip circumference, total physical activity, self-perceived health status, smoking status, alcohol consumption and use of dietary supplements).

**Figure 1:**
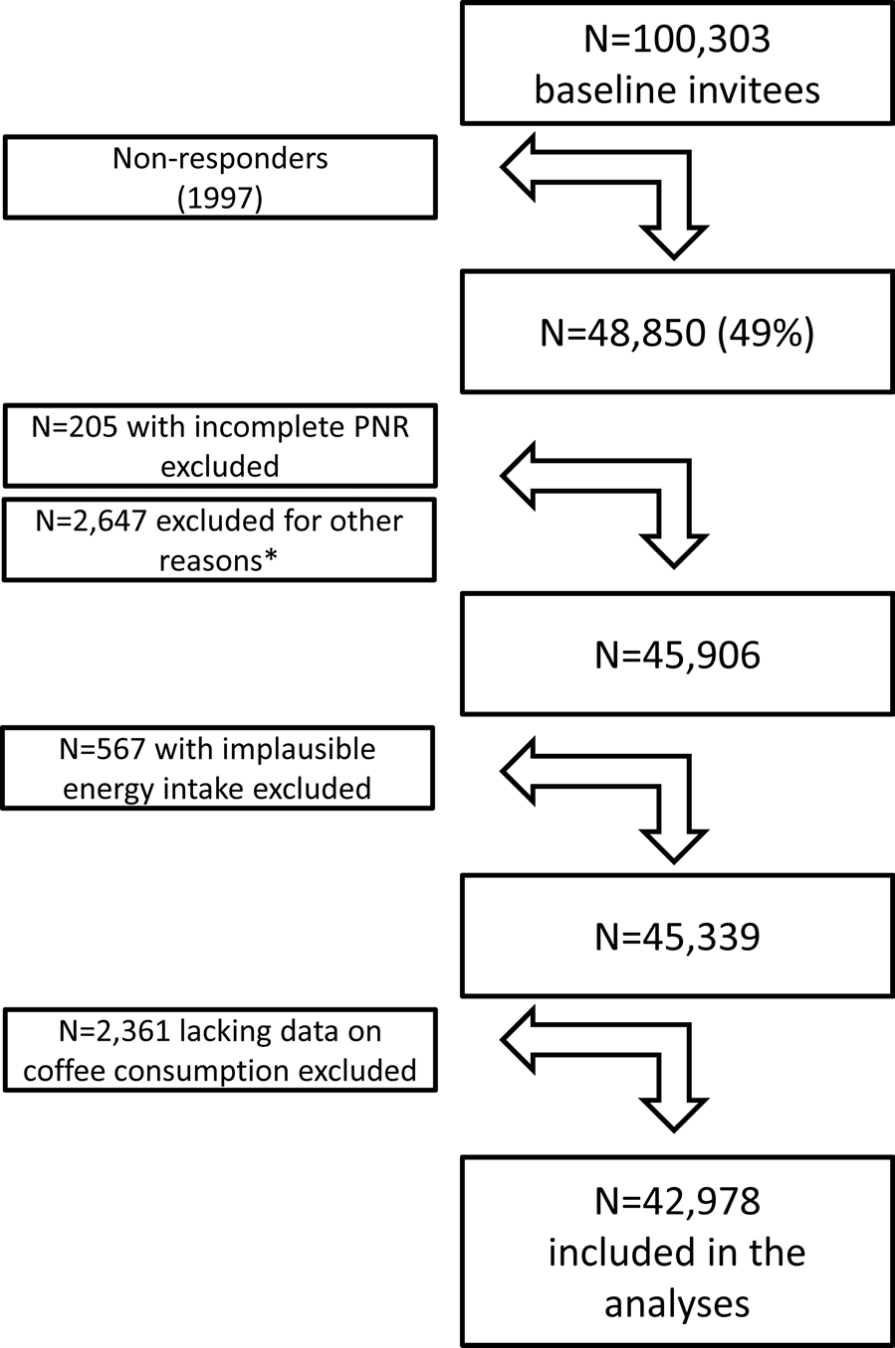
A flow chart describing the Cohort of Swedish Men (COSM). *Reasons for exclusions were: erroneous personal identification number, questionnaires not dated, erroneous dates of moving out of the study area or death, and a cancer diagnosis (except for non-melanoma skin cancer and only before the baseline questionnaire). Implausible energy intake was defined as ±3SD from the mean value of the log-transformed reported energy intake. Finally, individuals with lacking data on coffee consumption were excluded in the analyses. PNR: personal identification number.

Of the invited 100,303 men, 48,850 (49%) returned the questionnaire. The COSM is regarded as representative of Swedish men in this age range in terms of distribution of age, educational level and prevalence of overweight. From the baseline population, participants with incorrect or incomplete national registration numbers (n = 205) and those who reported an implausible energy intake (±3 SD of mean log-transformed energy, n = 567) were excluded. In addition, the following categories were excluded: men diagnosed with cancer other than non-melanoma skin cancer (n = 2,592) before baseline at 1 January 1998 or men who had passed away before 1 January 1998, as based on computerised linkage of the cohort to the National Cancer Register and the Population Register. Finally, we excluded an additional 2,361 men from the analyses in that these individuals had not stated their consumption of coffee even though non-use was a response possibility. Thus, the final sample for inclusion in the study was 42,978 men (Figure 1).

Written informed consent was obtained from the study participants and the study was approved by the Regional Ethical Review Board at Karolinska Institutet, Stockholm, Sweden.

### Dietary Assessment

Using a 96-item food frequency questionnaire (FFQ), dietary intake data were assessed at baseline (1997). Average frequency of consumption of coffee (number of cups consumed per day or week) during the previous year was recorded by the participants. Eight possible categories were used to report consumption frequency: never/seldom, 1–3 times per month, 1–2 times per week, 3–4 times per week, 5–6 times per week, once a day, 2 times a day and ≥3 times per day.

To calculate intake of nutrients the frequency of consumption of each food item was multiplied by the nutrient content of appropriate age-specific portion sizes obtained from the Swedish Food Agency Database [25]. Adjustment of nutrient intake using the residual method [26] was performed for total energy intake (2,200 kcal, which was the mean in men in the validation study of the FFQ [27], see below). The cup size was standardized to 200 ml (200 g) of coffee beverage. The FFQ-based information on coffee has been validated against data from fourteen 24-hour recall interviews over 1 year in a group of 248 men (Spearman’s correlation coefficient = 0.71) [28].

### Ascertainment of Fractures

Ascertainment was made of all first incident cases of any fracture (International Classification of Disease [ICD-10] codes S02, S12, S22, S32, S42, S52, S62, S72, S82, S92) and first incident cases of hip fracture (ICD-10 codes S720, S721, S722) registered in the National Patient Register [29] and regional hospital diagnosis registers between 1 January 1998 and 31 December 2010. Complete matching is enabled by use of the unique personal registration number provided to all Swedish citizens.

### Lifestyle and Comorbidity

Information about lifestyle variables, such as height (at age 20 years), weight, education, civil status, employment, alcohol consumption, smoking habits, cortisone use and physical activity, was obtained from the questionnaire.

According to five predefined categories, the participants recorded occupational physical activity, home/housework, walking/cycling duration per day and exercise duration per week during the past year before they were enrolled in the study [30]. To calculate total physical activity the reported time spent at each activity per day was multiplied by its typical energy expenditure requirements expressed in metabolic equivalents (MET). MET, expressed as kcal/kg/h, is the metabolic equivalent of sitting quietly for 1 hour [30]. The total sum of MET hours was used to create a MET (24-hour) score per day [30]. This variable was used in the statistical analyses. Validation of this method has been carried out and the Spearman correlation coefficient between the questionnaire and the activity records was 0.6 for the total activity score [30], [31]. Smoking habits were reported as smoking status (never, past, current). We calculated Charlson’s weighted comorbidity index [32], [33] based on diagnoses from the National Patient Register that had been registered before baseline.

### Statistical Analysis

For each participant, follow-up was calculated from January 1998 until the date of any fracture or hip fracture, date of death, date of leaving the study regions or the end of the study period (31^st^ of December 2010), whichever came first.

When individual data on coffee consumption were lacking (n = 2,361/45,339, corresponding to about 5% of the cohort), the participants affected were not retained as non-consumers in the analyses. It was found to be more appropriate to exclude them from the analyses because in a previous study [34] only 11% of the missing cases for coffee consumption were actually non-consumers. In the event individual data were lacking for covariates other than coffee consumption, multiple imputations were performed by applying the Markov Chain Monte Carlo multiple method to construct baseline values (all <5%).

To analyse the relation between consumption of coffee and risk of first fracture event (any fracture and hip fracture), crude- and multivariable-adjusted hazard ratios (HR) and 95% confidence intervals (CI) were estimated by Cox’s proportional hazards regression. Analyses were performed with coffee consumption as a continuous variable, with each unit corresponding to 200 ml (or one cup) of coffee. To compare our results with previous studies we also categorised coffee consumption into four categories (<1, 1, 2–3 and ≥4 cups daily). We further investigated the influence of very high coffee intake, i.e. ≥8 cups of coffee/day. For each category of coffee intake, age-adjusted failure curves (set at age 60 years corresponding to the mean baseline value) to illustrate fracture incidences were constructed by using the Kaplan-Meier method. Log-log plots for confirmation of the proportionality assumption were produced. The basic model used to estimate HRs included age. A multivariable model additionally included intakes of total energy, calcium, retinol, vitamin D, potassium, phosphorus, protein and alcohol, body mass index, height, physical activity (MET 24-hour score) (all continuous), intake of any vitamins, cortisone use, educational level (≤9, 12, >12 years, other), smoking status (never, former, current), previous fractures (yes or no) and Charlson’s comorbidity index (continuous) [32], [33]. Because intake of sleeping pills and 5α-reductase inhibitors or α1-receptor antagonists (indication mainly benign prostatic hyperplasia) only marginally affected the relations, these potential covariates were not included in the final multivariate model.

To analyse potential non-linear trends restricted cubic-spline Cox’s regression analyses were performed to flexibly model the associations between coffee intake and fracture risk [35]. Four knots placed at percentiles 5, 35, 65 and 95 of coffee consumption were used. The reference level was set to the lowest category of coffee intake (<1 cup of coffee/day). The results of these analyses are presented as smoothed curves with 95% CIs.

Statistical interactions between coffee consumption and calcium intake or age were assessed by creating a product term of the two and assessing whether this contributed to improved model fit by likelihood ratio testing. These interactions were further evaluated by performing stratified analyses using pre-defined cut-offs for calcium intake (below or above 800 mg/day, the recommended intake in Sweden) and for age (<50, 50–70 and ≥70 years).

All statistical analyses were performed using Stata version 11 (Stata Corp LP, Collage Station, TX, USA).

## Results

Baseline characteristics of the cohort are shown in Table 1. Forty-three per cent of the participants reported a daily consumption of 2–3 cups per day and 41% consumed 4 cups of coffee or more daily. The remaining 16% consumed 1 cup of coffee or less daily. In contrast, the consumption of tea was low. Among tea drinkers (57% of the participants in the cohort), 77% consumed 1 cup of tea or less per day.

**Table 1:** Baseline characteristics of study subjects by coffee consumption.

	Number of cups of coffee per day			
	<1 cup	1 cup	2–3 cups	≥4 cups
N (%)	2,318 (5.4)	4,514 (10.5)	18,366 (42.7)	17,780 (41.4)
Age at entry (yrs)	60.1 (9.6)	61.8 (9.6)	61.3 (9.8)	58.8 (9.4)
BMI at entry (kg/m^2^)	25.7 (3.7)	26.0 (3.4)	25.7 (3.3)	25.9 (3.3)
*Average intake per day* a				
Energy (kcal)	2,522 (879)	2,522 (879)	2,577 (775)	2,846 (852)
Calcium (mg)	1,416 (496)	1,423 (454)	1,458 (452)	1,495 (475)
Supplemental Calcium (mg)^b^	315 (485)	281 (380)	261 (332)	267 (394)
Total calcium^c^	1,457 (533)	1,453 (481)	1,485 (469)	1,518 (492)
Vitamin D (µg)	6.44 (3.01)	6.80 (3.60)	6.65 (2.97)	6.62 (2.80)
Retinol (µg)	1,751 (894)	1,804 (1019)	1,754 (872)	1719 (878)
Potassium (mg)	3,827 (703)	3,871 (680)	3,941 (640)	4,086 (669)
Protein (g)	102.0 (16.2)	102.2 (15.4)	102.1 (14.8)	102.7 (15.1)
Phosphorus (mg)	2043 (353)	2048 (333)	2068 (332)	2080 (349)
Alcohol (g)^d^	7.7 (22.3)	9.3 (19.7)	8.9 (20.0)	9.2 (21.4)
Coffee (g)^d^	84 (58)	212 (55)	552 (113)	999 (377)
Tea (g)^d^^,^ e	273 (485)	271 (352)	253 (294)	117 (295)
*Leisure time PA level, n (%)*				
1 (lowest)	214 (9.8)	500 (11.9)	1,627 (9.4)	1,873 (11.4)
2	404 (18.6)	714 (17)	2,913 (16.9)	2,598 (18)
3	801 (36.7)	1,517 (36)	6,508 (37.7)	5,963 (36.3)
4 (highest)	761 (34.9)	1,481 (35.2)	6,199 (35.9)	5,648 (34.4)
*Smoking status, n (%)*				
Current	394 (17.3)	795 (17.8)	3,778 (20.8)	5,684 (32.5)
Former	835 (36.6)	1,769 (39.6)	7,214 (39.8)	6,847 (39.1)
Never	1,051 (46.1)	1,901 (42.6)	7,149 (39.4)	4,986 (28.5)
Two or more Charlson’s comorbidities, n (%)	133 (5.7)	260 (5.8)	820 (4.5)	598 (3.4)
*Educational level, n (%)* f				
≤9 years	1,423 (61.7)	3,036 (67.5)	12,534 (68.5)	12,790 (72.1)
>9–12 years	1,363 (15.7)	652 (14.5)	2,532 (13.8)	2,377 (13.4)
>12 years	510 (22.1)	792 (17.6)	3,160 (17.3)	2,503 (14.1)
Other	110 (0.4)	21 (0.5)	77 (0.4)	65 (0.4)
Fracture before baseline, n (%)	327 (14.1)	515 (11.4)	2,184 (11.9)	2,176 (12.2)
Proscar use, n (%)	160 (2.6)	152 (3.4)	1,493 (2.7)	1,366 (2.1)
Cortisone use, n (%)	130 (5.6)	192 (4.3)	1,725 (4.0)	1, 732 (4.1)
Marital status, single, n (%)	566 (29.4)	910 (20.2)	3,023 (16.5)	3,013 (17.0)

Data shown are mean (SD) or n (%) where indicated.

a Energy-adjusted average nutrient data,

b Users of calcium supplements,

c All participants – mean values for calcium supplements were used,

d Median(SD),

e Number reporting consumption of tea: 22,942,

f Educational level “other” refers to vocational or other education.

**Figure 2:**
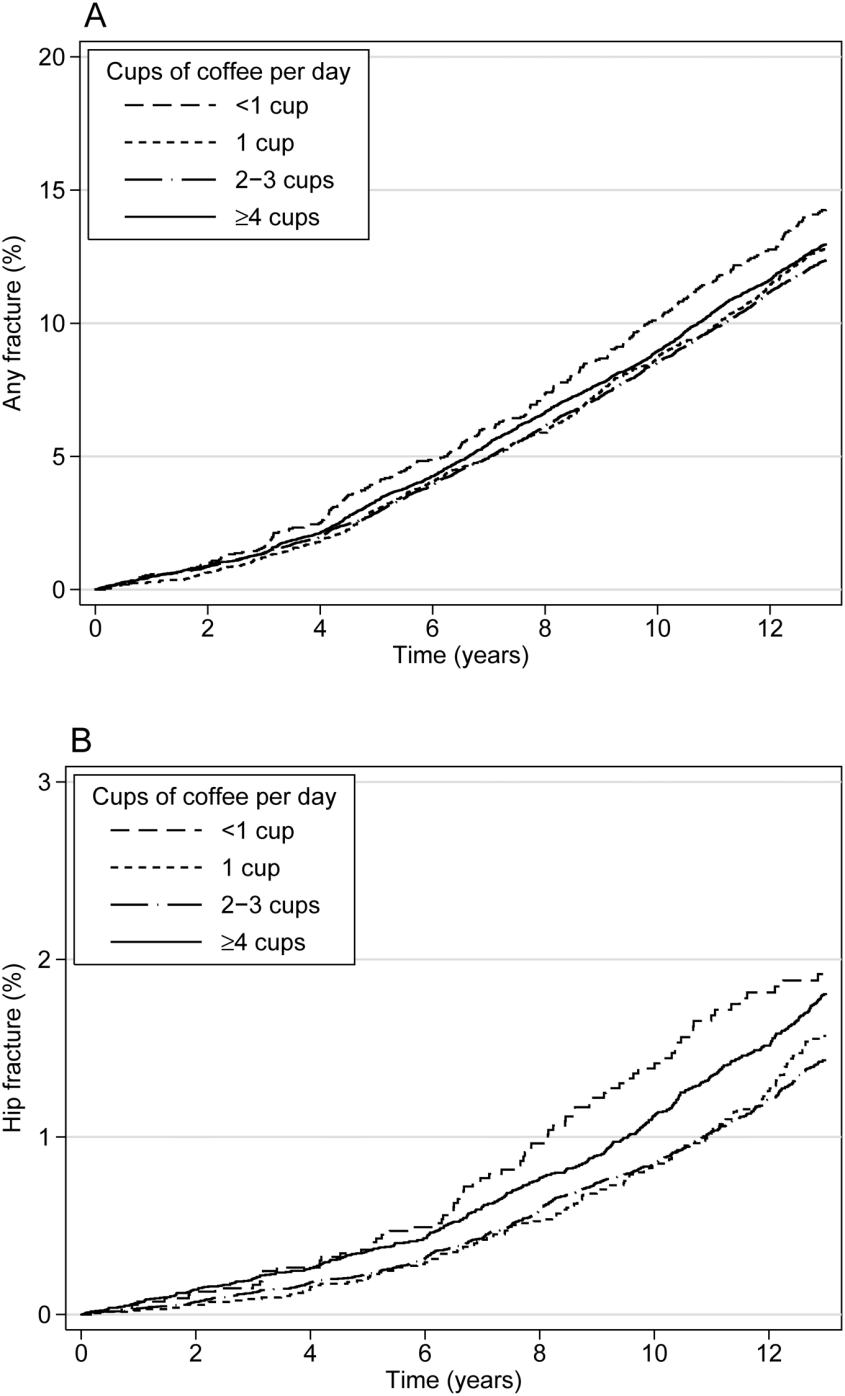
Fracture incidence in the Cohort of Swedish Men (COSM). Incidences of any fracture (Panel A) and hip fracture (Panel B) in relation to follow-up using Kaplan-Meier failure curves adjusted to age 60 for the four consumption categories of coffee (<1, 1, 2–3 and 4 cups or more per day).

A first fracture at any site was observed in 5,066 participants (11.8% of the cohort) during a median of 11.3 years of follow-up and 483,508 person years. During a median of 11.7 years of follow-up for hip fractures, there were 1,186 incident cases (2.8% of the cohort). For each category of coffee consumption (<1, 1, 2–3 and ≥4 cups per day), the age-adjusted incidence proportions of any fracture and hip fracture during follow-up are depicted in Figure 2. Smoking was more prevalent in the highest consumption category of coffee (≥4 cups daily) in comparison with categories with lower coffee consumption. Energy intake was somewhat higher in the category of participants drinking ≥4 cups of coffee daily than in the reference category.

When coffee exposure served as a continuous variable, no association was noted between increasing coffee consumption and rate of any fracture (HR 1.00; 95% CI 0.99–1.02) or hip fracture (HR 1.02; 95% CI 0.99–1.06) per 200 ml of coffee after multivariate adjustment. Similarly, no associations were found between increasing consumption and rate of any fracture or hip fracture after categorising coffee consumption into the four categories. HR for any fracture for men consuming ≥4 cups vs. <1 cup per day was 0.91 (95% CI 0.80–1.02) after multivariate adjustment. For hip fracture, the corresponding HR was 0.89 (95% CI 0.70–1.14) (Table 2).

**Table 2:** Coffee consumption and risk of any fracture and hip fracture in the Cohort of Swedish Men (COSM).

	Number of cups of coffee per day				
	<1 cup	1 cup	2–3 cups	≥4 cups	Coffee per 200 ml (Continuous)
Number of fractures	311	548	2,166	2,041	5,066
Person-years at risk	25,770	49,717	205,143	202,877	483,508
Rate/1000 person-years	12.1	11.0	10.6	10.1	10.5
Age-adjusted HR (95% CI)	1.00 (reference)	0.86 (0.75–0.98)	0.83 (0.74–0.94)	0.87 (0.77–0.98)	1.01 (0.99–1.02)
Adjusted HR (95% CI)^a^	1.00 (reference)	0.89 (0.78–1.02)	0.88 (0.78–0.99)	0.91 (0.80–1.02)	1.00 (0.99–1.02)
Number of hip fractures	78	135	526	447	1,186
Person-years at risk	26,959	51,807	213,260	211,106	503,131
Rate/1000 person-years	2.9	2.6	2.5	2.1	2.4
Age-adjusted HR (95% CI)	1.00 (reference)	0.76 (0.57–1.00)	0.73 (0.58–0.93)	0.85 (0.67–1.08)	1.03 0.99–1.06)
Adjusted HR (95% CI)^a^	1.00 (reference)	0.78 (0.59–1.03)	0.79 (0.62–1.00)	0.89 (0.70–1.14)	1.02 (0.99–1.06)

CI: confidence interval, HR: hazard ratio.

a Covariates included were: intake of total energy, calcium, retinol, vitamin D, potassium, phosphorus, protein, alcohol, body mass index, height, physical activity (MET 24-hour score) (all continuous), intake of any vitamins, cortisone use, educational level (≤9, 12, >12 years, other), smoking status (never, former, current), previous fractures (yes or no) and Charlson’s comorbidity index (continuous).

Moreover, even a consumption of eight cups of coffee or more per day, in comparison with <1 cup per day, was not associated with an increased risk of any fracture (HR 0.92; 95% CI 0.77–1.02) or hip fracture (HR 0.94; 95% CI 0.64–1.38). The prevalence of high consumption (≥8 cups) was 3.0%.

**Figure 3:**
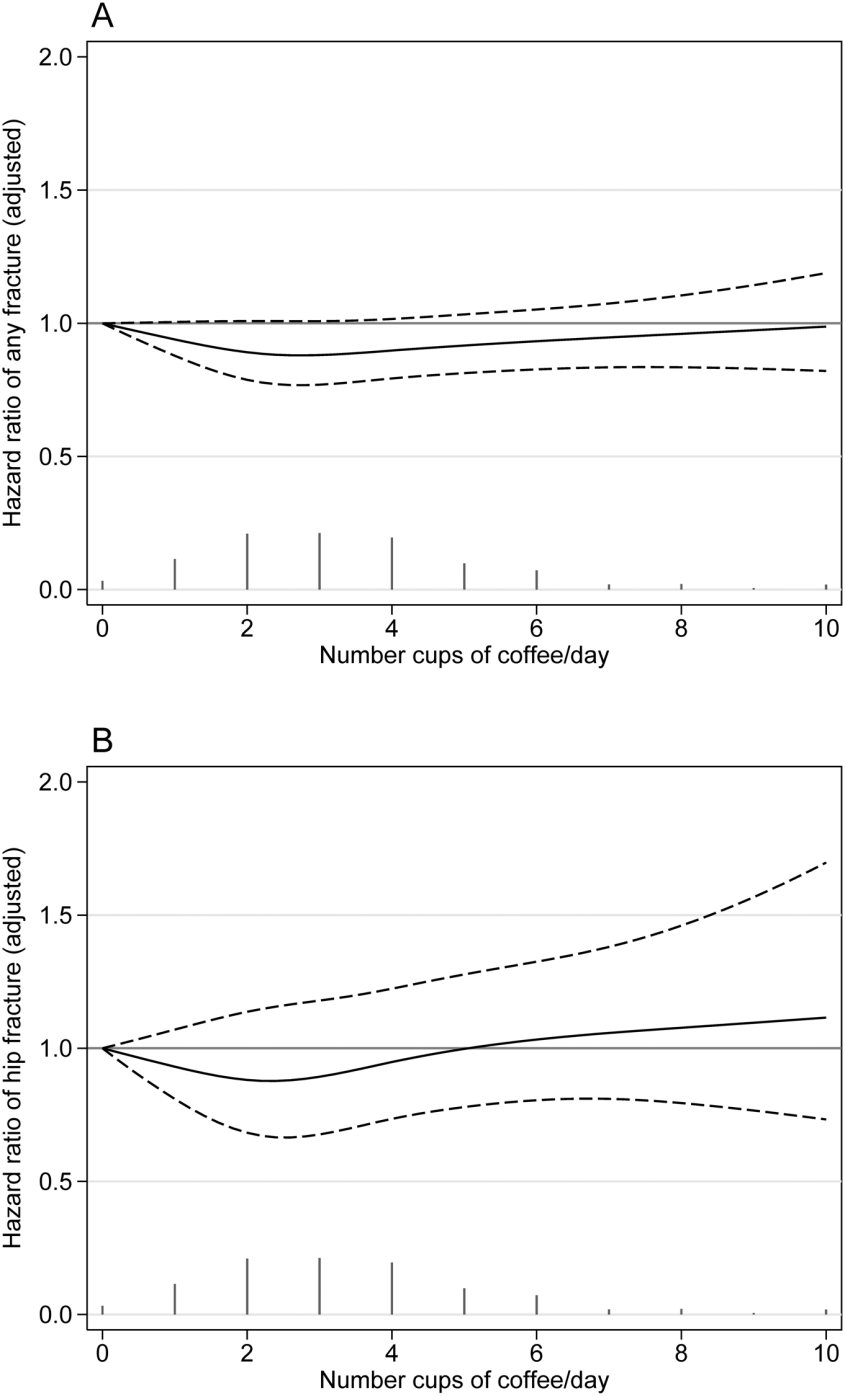
Association between coffee consumption and fracture risk. Multivariate-adjusted hazard ratios (HR) with 95% confidence intervals (CI) (dashed lines) of any fracture (Panel A) and hip fracture (Panel B) by coffee consumption. The vertical bars represent the distribution of coffee intake. The smoothed curves were fitted with a restricted cubic spline model with a consumption of <1 cup of coffee as the reference. Adjustments were made for intake of energy, protein, calcium, retinol, vitamin D, phosphorus, potassium and alcohol, body mass index, height, physical activity (MET-24 h score) (all continuous), intake of any vitamins, cortisone use, educational level (≤9, 12, >12 years, other), smoking status (never, former, current), previous fractures (yes or no) and Charlson’s comorbidity index (continuous).

As shown in Figure 3, no non-linear associations between consumption of coffee and incidence of any fracture or hip fracture could be observed.

We observed no indication of the presence of effect modification by calcium intake on the association between coffee consumption and fracture risk (P = 0.46 for any fracture and P = 0.28 for hip fracture). There was no indication of an interaction between age and coffee consumption (P = 0.11 for any fracture and P = 0.28 for hip fracture). The stratified analyses gave similar results as the full analysis (not shown).

## Discussion

No significant association was found between consumption of coffee and incidence of fractures in this large prospective cohort of Swedish men. Furthermore, this result was not modified by either calcium intake or age.

The results from this investigation in men are in line with the results in our recent study of a large cohort of Swedish women. In this study a coffee consumption of ≥4 cups daily was associated with a decrease in BMD, but this decrease did not translate into an increased risk of fractures [20]. We previously observed lower BMD of the proximal femur with higher consumption of coffee in men [21]. Epidemiological research in men regarding coffee consumption and risk of fracture is rather scarce. The male part of the multicentre MEDOS case-control study by Kanis *et al*, 1999 [36], collected 730 hip fracture cases and 1,132 controls from Southern Europe. In this study no association between past coffee consumption or caffeine intake recalled after the fracture event and the risk of hip fracture was demonstrated. In a study by Kiel *et al* [13], a part of the Framingham cohort was investigated to assess intake of caffeine and risk of hip fracture. In males, caffeine intake corresponding to two cups of coffee or four cups of tea was associated with an increased risk of hip fracture, although this was not statistically significant and based on a limited number of fractures (n = 22). In a large Norwegian cohort study including over 20,000 men with mean age 47 years, dietary factors in relation to hip fracture incidence were examined [14]. With 11 years of follow-up and 56 incident hip fracture cases, the authors did not observe an association between coffee intake and fracture risk. In a prospective cohort of Swedish middle-aged men (n = 7,495) followed for 30 years and aiming at identifying risk factors for hip fracture, Trimpou *et al* [15] found that coffee consumption was associated with a lower risk of hip fracture (n fractures = 451). There seemed not to be a dose-response effect in the unadjusted analysis and in the multivariate analysis, coffee consumption was dichotomized into any consumption and no consumption. The authors state that this association could be explained by adverse characteristics among those who did not drink coffee. The three cohort studies with information on estimates in men have been summarized in a recent meta-analysis [16], suggesting a decreased risk of hip fracture with increasing coffee consumption. The analysis is greatly influenced by the Trimpou study [15] with a weight of 76%. To summarise, the few available cohort studies in men have limitations because of few fractures [13], [14], that the only exposure considered was caffeine as a pooled estimate, *i.e.* the exposure calculation included not only coffee [13], or that coffee consumption was considered as any vs. no consumption [15]. The present study exceeds by far the total number of hip fractures in previous cohort studies and also had the possibility to study a large number of fractures of any type (we observe 1,186 hip fractures and 5,066 fractures of any type).

### Strengths and Limitations

One of the most important strengths of our study is that we had the opportunity to collect data from a large population-based cohort of middle-aged and elderly men during a mean follow-up of 11.3 years. Such a follow-up is sufficiently long to observe an adequate number of fractures. Because all fractures were identified by the use of registers, we believe that the risk of not having detected men with a fracture during follow-up is small. There was considerable variation in consumption of coffee in this cohort with a large number of participants consuming high amounts of coffee, which improves the chances of detecting associations. In this context it should be noted that the consumption of decaffeinated coffee is very low in Sweden (<1%) [37]. Moreover, we did not focus on intake of caffeine, but on consumption of coffee, which might be another advantage in that several studies have indicated that tea could have a positive influence on BMD [38]–[40] and fracture risk [36], probably because of the fluoride, phytoestrogen or antioxidant content of tea [39]. Finally, it should be possible to generalise our results to all men in Sweden because the participants well represent the source population [41].

We also acknowledge a number of potential limitations. Because this investigation is based on data from one single FFQ, some degree of error in the exposure measurement cannot be excluded. Attenuation of a true association is likely in that the potentially resulting misclassification probably would be non-differential. Fractures associated with high trauma were not excluded because a comparable increased risk of both low- and high-trauma fracture with decreasing bone density in the elderly has been indicated [42]. However, there has been discourse as to whether inclusion of both high and low impact fractures will result in a lower risk estimate compared with low trauma fractures only [43]. Despite controlling for known major risk factors for fractures, including comorbidity, it is still possible that residual confounding could have influenced the results of this study. For instance, we could not adjust for vitamin D status or sunlight exposure in the current study. However, we have previously shown that the effect of coffee intake on BMD was not stronger among women with low vitamin D status [20]. The importance of the dietary source of protein (*i.e.* animal or vegetable) on the association between coffee consumption and fracture could not be assessed in the present study. There is to date no consensus on the relation between dietary protein and fracture risk [44] but recent systematic reviews and meta-analyses suggest that the postulated dietary acidic load exaggerated by protein intake does not have a causal effect on bone health [45], [46]. Carriers of a genetic variant of the vitamin D receptor might be more vulnerable toward the effects of caffeine on bone [47]. In fact, results from our previous study suggest that genetically determined differences in caffeine metabolism might be of importance for how BMD is affected by coffee/caffeine [21]. However, in this study genotyping of the participants was not performed. Furthermore, we did not have the possibility to measure BMD in this cohort. Such a measurement might have been of interest because in an earlier study we obtained evidence of a modest decrease in BMD of the proximal femur among elderly men (aged 72 years) drinking 4 cups of coffee or more per day [21]. In the context of previous research, in which no association between coffee consumption and fracture risk has been observed, the small impact in the relation between BMD and coffee does not seem to influence the risk of fracture among men on the population level. Intervention on causes of fracture other than coffee consumption would probably have a larger impact on fracture incidence.

## Conclusion

In conclusion, we did not observe an increased risk of osteoporotic fractures in this large cohort of Swedish middle-aged and elderly men. Calcium intake did not influence risk for fracture of any type or hip fracture.
